# Anxiety-like behavior and anxiolytic treatment in the Rett syndrome natural history study

**DOI:** 10.1186/s11689-022-09432-2

**Published:** 2022-05-14

**Authors:** Caroline B. Buchanan, Jennifer L. Stallworth, Aubin E. Joy, Rebekah E. Dixon, Alexandra E. Scott, Arthur A. Beisang, Timothy A. Benke, Daniel G. Glaze, Richard H. Haas, Peter T. Heydemann, Mary D. Jones, Jane B. Lane, David N. Lieberman, Eric D. Marsh, Jeffrey L. Neul, Sarika U. Peters, Robin C. Ryther, Steve A. Skinner, Shannon M. Standridge, Walter E. Kaufmann, Alan K. Percy

**Affiliations:** 1 Greenwood Genetic Center, 106 Gregor Mendel Circle, Greenwood, SC 29649 USA; 2 Gillette Children’s Hospital, St. Paul, MN USA; 3 Children’s Hospital Colorado, University of Colorado at Denver, Denver, CO USA; 4 Baylor College of Medicine, Houston, TX USA; 5 Rady Children’s Hospital-San Diego, University of California, San Diego, CA USA; 6 Rush University Medical Center, Chicago, IL USA; 7 UCSF Benioff Children’s Hospital of Oakland, Oakland, CA USA; 8 Civitan International Research Center, University of Alabama at Birmingham, Birmingham, AL USA; 9 Boston Children’s Hospital, Harvard Medical School, Boston, MA USA; 10 Children’s Hospital of Philadelphia, Perelman School of Medicine at the University of Pennsylvania, Philadelphia, PA USA; 11 Vanderbilt Kennedy Center, Vanderbilt University Medical Center, Nashville, TN USA; 12 Washington University School of Medicine in St. Louis, St. Louis, MO USA; 13 Division of Neurology, Cincinnati Children’s Hospital Medical Center, Cincinnati, OH USA; 14 Department of Pediatrics, University of Cincinnati College of Medicine, Cincinnati, OH USA; 15 University of South Carolina School of Medicine, Columbia, SC USA; 16 Emory University School of Medicine, Atlanta, GA USA

**Keywords:** Rett syndrome, Natural history studies, Anxiety, Anti-anxiety agents, Methyl-CpG-binding protein 2

## Abstract

**Background:**

Rett syndrome (RTT) is a neurodevelopmental disorder most often related to a pathogenic variant in the X-linked *MECP2* gene. Internalizing behaviors appear to be common, but standard methods of diagnosing anxiety are not readily applied in this population which typically has cognitive impairment and limited expressive language. This study aims to describe the frequency of anxiety-like behavior and anxiolytic treatments along with associated clinical features in individuals with RTT.

**Methods:**

Parental reports and medication logs provided data from 1380 females with RTT participating in two iterations of the multicenter U.S. RTT Natural History Study (RNHS) from 2006 to 2019.

**Results:**

Most participants with RTT (77.5%) had at least occasional anxious or nervous behavior. Anxiety was reported to be the most troublesome concern for 2.6%, and within the top 3 concerns for 10.0%, of participants in the second iteration. Parents directly reported treatment for anxious or nervous behavior in 16.6% of participants in the second iteration with most reporting good control of the behavior (71.6%). In the medication logs of both RNHS iterations, the indication of anxiety was listed for a similar number of participants (15% and 14.5%, respectively). Increased use of anxiolytics and selective serotonin reuptake inhibitors (SSRIs) was related to more frequent anxiety-like behaviors (*P* < 0.001), older age (*P* < 0.001), and mild *MECP2* variants (*P* = 0.002).

**Conclusion:**

Anxiety-like behavior is frequent at all ages and is a significant parental concern in RTT. Older individuals and those with mild *MECP2* variants are more likely to be treated with medications. Better diagnosis and treatment of anxiety in RTT should be a goal of both future studies and clinical care.

**Trial registration:**

NCT00299312 and NCT02738281

## Background

Rett syndrome (RTT; OMIM 312750) is a genetic neurodevelopmental disorder that occurs in approximately 1 in 10,000 females [13]. Intellectual disability and communication and motor deficits occur after a period of regression with recovery or stabilization in early childhood [2, 11]. While individual presentations can vary greatly, diagnostic criteria for RTT include a period of regression plus 4 characteristic neurologic features: partial or complete loss of acquired hand skills, partial or complete loss of acquired spoken language, absent or abnormal gait, and hand stereotypies [22]. If all 4 main criteria are met, the diagnosis is considered classic (or typical) RTT while individuals with variant (or atypical) RTT meet at least 2 of the 4 main criteria plus 5 of 11 supportive criteria. Behavioral difficulties, such as inappropriate fear and abrupt mood changes, though not included in the diagnostic criteria, appear to be common and have been recognized since the early descriptions of the disorder [1, 3, 8, 19]. The neurologic features of RTT are typically related to a pathogenic variant in the X-linked gene encoding the methyl-CpG-binding protein 2 (MeCP2), a transcriptional regulator involved in synaptic development and maintenance of neuronal circuitry [2]. While genotype-phenotype correlations are limited, a few of the more than 500 *MECP2* pathogenic variants in RTT are known to correlate with disease severity [14]. Increased age also correlates with increased disease severity [11, 14].

The US RTT Natural History Study (RNHS) is a longitudinal, observational study of the largest cohort of individuals with the disorder. The data collected included age, diagnosis (typical vs. atypical), *MECP2* variant, and a variety of specific clinical features, including two scales of clinical severity. Anxiety-specific assessments and psychiatric diagnoses were not collected in the RNHS. However, the first iteration of the RNHS contained medication logs providing information on treated behavioral disorders (including anxiety) and the second iteration, in addition to medication logs, collected parent-reported severity of anxious or nervous behavior, and medication use and effectiveness for anxiety.

Anxiety is considered an internalizing behavior, along with depressed mood, obsessive-compulsive behaviors, and somatic complaints [6, 18, 30]. Although the exact prevalence of anxiety in RTT is unknown [1, 3, 8, 19], we previously reported that internalizing behaviors, as defined by the Mental Health/Well-being (MH) scale from the Child Health Questionnaire–Parent Form 50 (CHQ-PF50) as feeling lonely, feeling like crying, acting upset or acting nervous [17], are common and clinically significant in RTT in the first iteration of the RNHS. Indeed, scores on the CHQ MH in classic RTT were comparable to those in individuals with a range of psychiatric diagnoses and worse than in patients with epilepsy and in the general population. Internalizing behaviors were more severe in participants with mild *MECP2* pathogenic variants (R133C, R294X, R306C, and C-terminal truncations) when compared to participants with either moderate (T158M and other point mutations) or severe pathogenic variants (R106W, R168X, R255X, R270X, early truncations, large deletions, and splice site) [5]. Another study that examined the profiles of anxious behavior in girls with RTT similarly reported that higher anxiety scores predominantly occurred in children with less severe neurologic impairment and were associated with a lower quality of life [3].

Existing diagnostic and assessment tools for anxiety and other internalizing behaviors are often inappropriate in RTT because of the cognitive impairment and limited verbal communication of this population. Further complicating the recognition of anxiety in individuals with RTT is the common presence of anxiety-like behavior, such as worsening of hyperventilation and breath holding, in situations that would not be expected to induce anxiety. Other manifestations of anxiety in RTT may include inability to relax, inconsolable crying, tenseness, trembling, screaming episodes, avoidance of eye contact, and social withdrawal [3, 8, 9, 12, 15, 19–21, 25, 27, 28]. Barnes et al. [3] demonstrated the feasibility of evaluating different subtypes of anxiety behaviors in children with RTT using the Anxiety, Depression, and Mood Scale (ADAMS) for individuals with intellectual disability. Nevertheless, of the five ADAMS subscales, only the Social Avoidance subscale showed the range of psychometric properties suitable for clinical and research applications in this population.

In spite of the general impression that anxiety-like behavior is common in individuals with RTT, no systematic studies of anxiolytics use have been reported in this population and guidelines for clinical management of anxiety in RTT do not exist. Selective serotonin reuptake inhibitors (SSRIs) have been suggested for the treatment of anxiety and abnormal mood while benzodiazepines have been recognized as a potential therapy for breathing irregularities [7]. We conducted an exploratory analysis of individuals being treated with anxiolytic medication (e.g., SSRIs); in line with current perspectives on anxiety in neurodevelopmental disorders, treated individuals had more severe profiles of internalizing behaviors than their non-treated counterparts [5].

Given the apparent high frequency of anxiety-like behaviors in individuals with RTT, the significant impact anxiety can have on quality of life, the shortage of appropriate assessment tools for this mostly nonverbal population, and the lack of guidelines on the clinical management of anxiety in RTT, the goal of this study was to provide the first large-scale description of anxiety-like behavior in RTT using data from the RNHS. In this investigation, we characterize individuals with RTT reported to have anxious behavior or being treated for anxiety to address the current gap in knowledge.

## Methods

### Participants

**Fig. 1:**
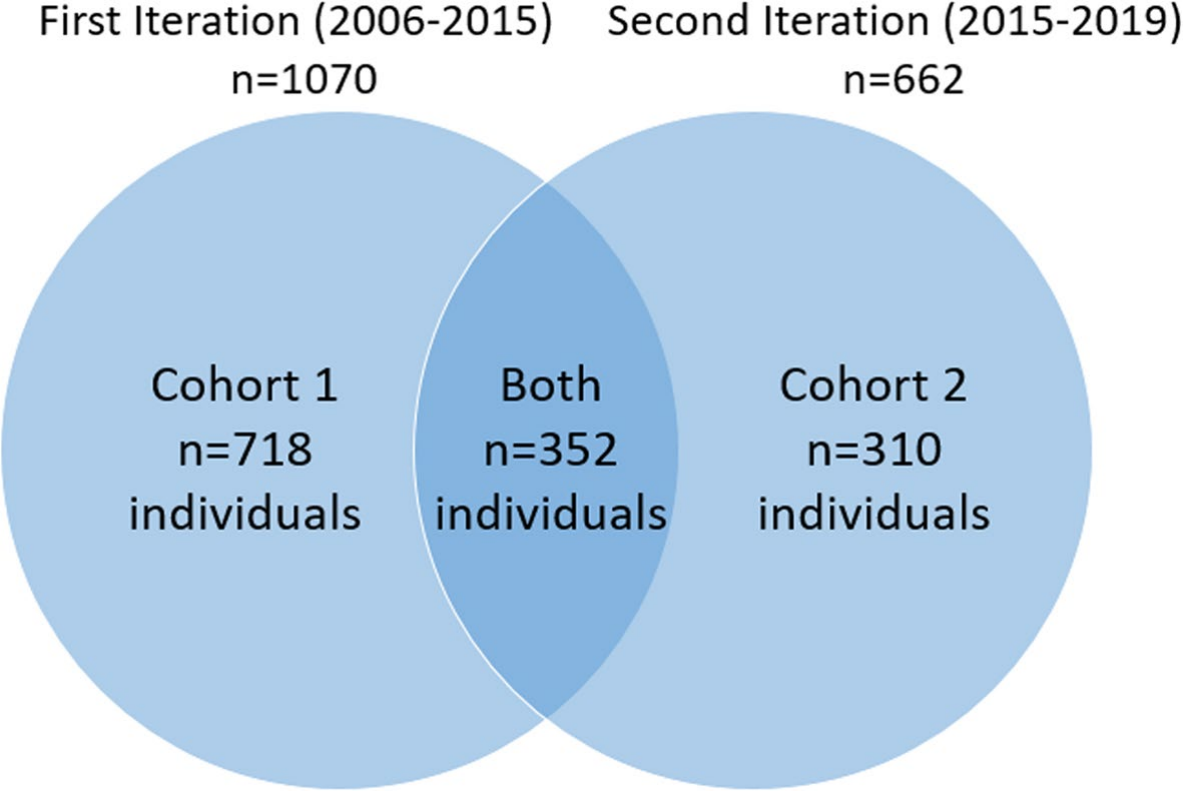
Rett syndrome natural history study (RNHS) participants selected for analysis (*n* = 1380). Both = subjects participating in both the first and second iterations; cohort 1 = subjects only participating in the first iteration; cohort 2 = subjects only participating in the second iteration

Participants were recruited through the multicenter RNHS to provide suitable longitudinal information that could serve as the foundation for conducting translational research including clinical trials. Assessments were conducted every 6 to 24 months by clinicians at one of eight sites in the USA from 2006 to 2015 (first iteration) and at 1 of 14 sites from 2015 to 2019 (second iteration). Data from 1380 females with RTT (718 in the first iteration only, 310 in the second iteration only, and 352 in both iterations) were included (Fig. 1). Medication log data from the first iteration were compared with parental report of anxiety-like behavior from the second iteration. Participants from both iterations allowed confirmation of parental report accuracy. Only baseline measures from each iteration were analyzed for this report. All female participants with a diagnosis of RTT (typical and atypical), regardless of *MECP2* pathogenic variant status, were considered for inclusion in the analyses presented here. For this analysis, data were excluded from males, participants with a non-RTT diagnosis, and participants missing significant baseline data. The RNHS consortium is part of the Rare Diseases Clinical Research Network (RDCRN), an initiative of the Office of Rare Diseases Research, National Center for Advancing Translational Sciences, National Institutes of Health.

### Human studies approval

Parental consent for study conduct and publication of results was obtained prior to entry into the study for all participants. Each participating institution retained institutional review board approval for the implementation of this study protocol and consent form (ClinicalTrials.gov identifiers: NCT00299312 and NCT02738281).

### Diagnosis

Diagnoses of RTT were based on published diagnostic guidelines, whether classic (typical), variant (atypical), or other related phenotypes by a RNHS site Principal Investigator [22].

### MECP2 pathogenic variants

*MECP2* variants were reported from clinical laboratory testing. We categorized pathogenic variants as severe (R106W, R168X, R255X, R270X, early truncations, large deletions, and splice site), moderate (T158M and other point mutations), or mild (R133C, R294X, R306C, and C-terminal truncations) based on prior reports of genotype-phenotype correlations [4, 11, 23, 29]. Participants with clinical RTT without a *MECP2* variant or with variants in exon 1 were excluded from analyses involving *MECP2* severity.

### Measures

#### Clinical severity scale (CSS)

The CSS is a clinician-completed questionnaire that uses a Likert-type scale to measure clinical features common in RTT, including age at onset of regression, somatic and head growth status, motor, communication, and RTT behaviors/other neurologic symptoms. Each item is ranked from either 0–4 or 0–5, with higher scores indicating greater clinical severity (range 1–58).

#### Motor behavioral assessment (MBA)

The MBA is a clinician-completed questionnaire for RTT that uses a Likert-type scale to score multiple items based on severity from 0 to 4, with a maximum total score of 148 (first iteration) or 136 (second iteration, with 3 previously low-scoring items removed). The scale includes measures of behavioral/social assessment, orofacial/respiratory assessment, and motor assessment/physical signs. A higher score indicates greater clinical severity.

### Anxiolytic and/or SSRI treatment

The first iteration of the RNHS did not include direct parental assessment of anxiolytics use but the second iteration explicitly asked if the child was prescribed medication for being anxious or nervous. Both iterations of the RNHS collected comprehensive data regarding developmental and medical history, including medication use and indication. Medication logs from both iterations were reviewed for the indication of “anxiety” entered as either free text or as a SNOMED code, regardless of the type of medication associated with that entry. Medication logs were also reviewed for reported use of any SSRI, regardless of the indication associated with that entry.

### Anxious behavior

The first iteration of the RNHS did not include specific questions about anxious behavior but the second iteration explicitly asked how frequently the child was anxious or nervous (with results on a scale of 0–4), whether the child was medicated for being anxious or nervous and, if yes, was the behavior well or poorly controlled. Parents were also asked to choose from a list of common features of RTT and select the top 3 features that have had the greatest impact on their child’s quality of life in the past 6 months.

### Analytical strategy

Data from the second iteration of the RNHS constituted the primary source for the analyses, which included frequency of anxious/nervous and other abnormal behaviors, frequency and effectiveness of treatment for anxiety, drug type used to treat anxiety in RTT, and clinical characteristics of participants. Data from the first iteration of the RNHS were used as a potential source of convergent evidence for profiles of individuals with RTT treated pharmacologically for anxiety. Comparisons of direct report and medication logs for overlapping and non-overlapping subgroups of the second iteration were employed to assess comparability of first and second iteration datasets and accuracy of parental report of anxiety-like behavior.

### Statistical analyses

Descriptive statistics were used for characterizing most parameters, with continuous variables described in terms of means, standard deviations (SD), and ranges and categorical variables mainly as frequencies. Groups’ age and clinical severity, based on CSS or MBA total scores, were compared using the Mann-Whitney *U* or Kruskal-Wallis tests. Parent-reported frequency of anxious behavior in different groups was compared by Chi-square test or Fisher’s exact test. Frequency of other parameters and categorical variables, such as race, ethnicity, RTT diagnosis, *MECP2* severity, parental report of anxiolytic use, medication log report of anxiolytic use, and medication log report of SSRI use, were also analyzed by these tests. Two-sided *P* values less than 0.05 were considered significant; Bonferroni adjustments for multiple comparisons were used for analyses where appropriate. The analyses were performed using SPSS version 24.

### Characteristics of the study population

Figure 1 displays the number of participants across the years of the first (2006–2015) and second (2015–2019) iterations of the RNHS, including the number of individuals unique to each study and those participating in both iterations. Table 1 displays the clinical features of the participants. Race and ethnicity differ between the study iterations, though most participants (> 85%) are White and non-Hispanic. Participants in the second iteration of the RNHS were older than the first iteration and displayed higher clinical severity scores, as expected from earlier studies. Older age was expected in the second iteration given the overlap of participants. Unexpectedly, when overlapping participants were removed from analysis, on average, participants in the second iteration were older than participants in the first iteration but had similar clinical severity. The type of RTT diagnosis (typical or atypical) and *MECP2* variant severity did not differ between the first and second iteration.

**Table 1:** Baseline clinical characteristics of participants from the Rett syndrome natural history study (RNHS) analyzed in this study

**Characteristic**	**Category**	**First iteration** (*n* = 1070)		**Second iteration** (*n* = 662)
**Race**^1^**	White	88%		92%
	Black or African American	7%		5%
	American Indian or Alaska Native	3%		1%
	Asian	5%		5%
	Native Hawaiian or Pacific Islander	(Not assessed)		1%
Ethnicity*	Not Hispanic, Latino, or Spanish Origin	85%		87%
	Hispanic, Latino, or Spanish Origin	15%		11%
**RTT diagnosis**	Classic (typical)	87%		89%
	Variant (atypical)	13%		11%
***MECP2*** **severity**	Mild	32.5%		35.0%
	Moderate	16.7%		17.0%
	Severe	50.8%		47.9%
Age***		9.1 years ± 8.8 (range 1–66)		15.2 years ± 10.7 (range 1–58)
CSS total***		22.2 ± 7.8		23.8 ± 8.3
MBA total**		46.9 ± 15.0		48.8 ± 13.8
		**Cohort 1** (*n* = 718)	**Both studies**^**a**^ (*n* = 352)	**Cohort 2** (*n* = 310)
**RTT diagnosis**	Classic (typical)	83.4%	93.5%***	83.2%
	Variant (atypical)	16.6%	6.5%***	16.8%
***MECP2*** **severity**	Mild	32.4%	32.7%	37.7%
	Moderate	16.4%	17.1%	17.0%
	Severe	51.1%	50.1%	45.3%
**Age**		9.22 ± 8.6 (range 1–66)	8.84 ± 9.1 (range 1–48)	12.56 ± 10.7*** (range 1–52)
**CSS total**		22.92 ± 7.9	20.79 ± 7.4***	22.18 ± 8.4
**MBA total**		49.01 ± 15.2	42.64 ± 13.6***	47.84 ± 14.5

*Both* Subjects participating in both the first and second iteration, *Cohort 1* Subjects only participating in the first iteration, *Cohort 2* Subjects only participating in the second iteration, *CSS* Clinical Severity Scale, *First iteration* Subjects enrolled 2006–2015, *MBA* Motor Behavioral Assessment, *Mild MECP2 severity* R133C, R294X, R306C, and C-terminal truncations, *Moderate MECP2 severity* T158M and other point mutations, *RTT* Rett syndrome, *Severe MECP2 severity* R106W, R168X, R255X, R270X, early truncations, large deletions, and splice site, *Second iteration* Subjects enrolled 2015–2019

^1^More than one option could be selected so total > 100%; ***P < 0.001, **P < 0.01, *P < 0.05 compared to first iteration or cohort 1

^a^Baseline data from first iteration

## Results

### Frequency of anxiety-like behavior

**Fig. 2:**
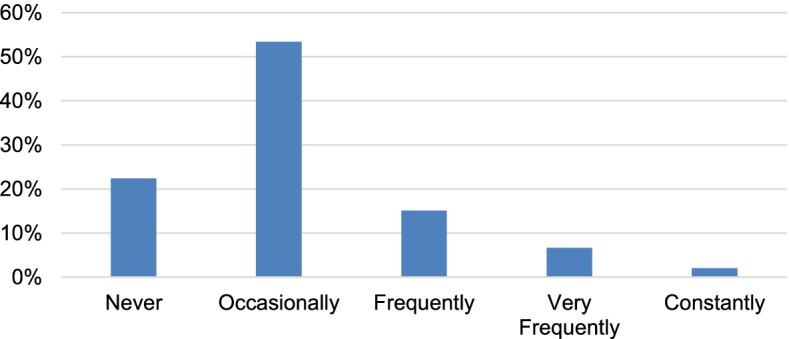
Responses to: How often is your child anxious or nervous? From 662 participants in the second iteration of the RNHS. First iteration = subjects enrolled 2006–2015; second iteration = subjects enrolled 2015–2019

During the second iteration of the RNHS, data were obtained specifically asking parents to report how often their child was anxious or nervous. A majority of participants (77.5%) were reported to be at least occasionally anxious/nervous, with 24.1% being reported as anxious/nervous frequently, very frequently, or constantly (Fig. 2). Frequency of being anxious or nervous was not different based on type of RTT diagnosis (typical or atypical, *P* = 0.171) or whether participants were enrolled in both iterations or only in the second (*P* = 0.406). Frequency of being anxious showed no correlation with age or clinical severity based on MBA total score. Increased frequency of anxious behavior correlated with lower severity based on CSS total score, but the magnitude of the association was negligible (Spearman’s rho = − 0.151, *P* < 0.01). Participants with mild *MECP2* variants had higher frequency of anxious behavior than participants with more severe variants (*P* < 0.001).

### Severity of anxiety-like behavior

**Fig. 3:**
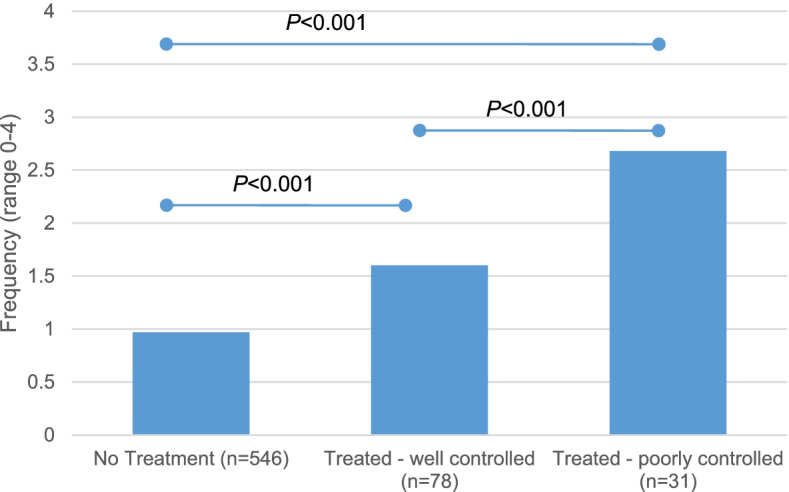
Frequency of anxious/nervous behavior based on direct report of anxiolytic treatment. Frequency values: 0 = “never”, 1 = “occasionally”, 2 = “frequently”, 3 = “very frequently”, 4 = “constantly”

Severity was assessed in the second iteration through parental report of anxious/nervous behavior as a major concern and through direct parental report of treatment. Specifically, the second iteration of the RNHS asked parents to select “the three biggest problems” affecting their child’s quality of life in the past six months from a list including both behavioral and medical complications of RTT: anxiety was the greatest concern for 2.6% and was within the top 3 concerns for 10% of participants (data not shown). Parents also reported whether their child displayed certain problem moods or behaviors, and whether those required treatment. Being anxious/nervous was the most common behavioral problem reported and treated, with a majority of those reporting good control of symptoms with treatment (Table 2). The frequency of anxious/nervous behavior was greater in those participants reporting treatment and in those with reported poor control (Fig. 3). Participants treated for anxiety by direct parental report were older than those without treatment (*P* < 0.001). In addition, participants with good control of anxious/nervous behavior were also older than those with poor control or no treatment (*P* < 0.001). Parental report of treatment frequency differed by *MECP2* variant severity (*P* < 0.001); participants with mild variants were more than twice as likely as participants with severe variants to report treatment for being anxious or nervous (23.5% vs 10.1%). The percent of participants reporting treatment for being anxious did not differ based on RTT diagnosis type (typical or atypical) (*P* = 0.795), study enrollment (both or second iteration only, *P* = 0.356), or clinical severity based on CSS or MBA total scores (*P* = 0.186, *P* = 0.993).

**Table 2:** Rett syndrome natural history study (RNHS) participants' direct report of treatment for behavioral problems (*n* = 655)

Is your child treated for:	No	Yes, my child is treated for this and it is well controlled	Yes, my child is treated for this but it is poorly controlled
Being anxious or nervous	83.4%	11.9%	4.7%
Being sad, miserable, or uncomfortable	87.8%	8.1%	4.1%
Rapid changes in mood	91.0%	6.1%	2.9%
Being irritable	91.8%	5.5%	2.7%
Screaming episodes	93.4%	4.1%	2.4%
Self-abusive behaviors	95.9%	2.9%	1.2%
Being aggressive and abusive to others	96.6%	2.1%	1.2%
Being excessively active	98.5%	1.1%	0.5%
Having a low level of activity	99.2%	0.3%	0.5%

### Frequency of anxiolytic use by medication logs

**Fig. 4:**
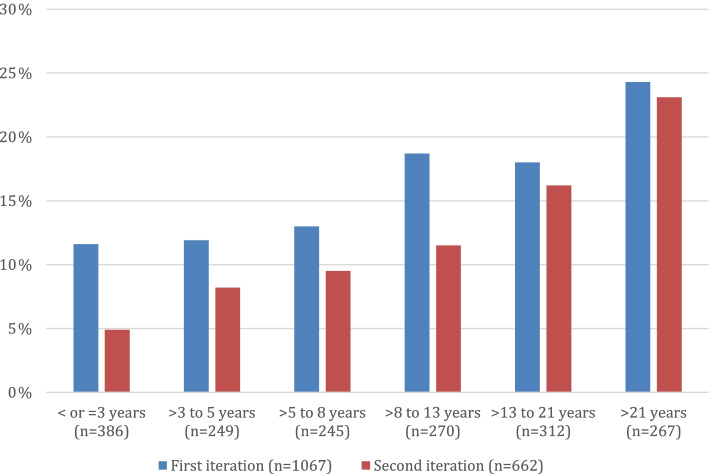
Frequency of anxiolytic use reported in medication logs by age group. First iteration = subjects enrolled 2006–2015; second iteration = subjects enrolled 2015–2019

**Fig. 5:**
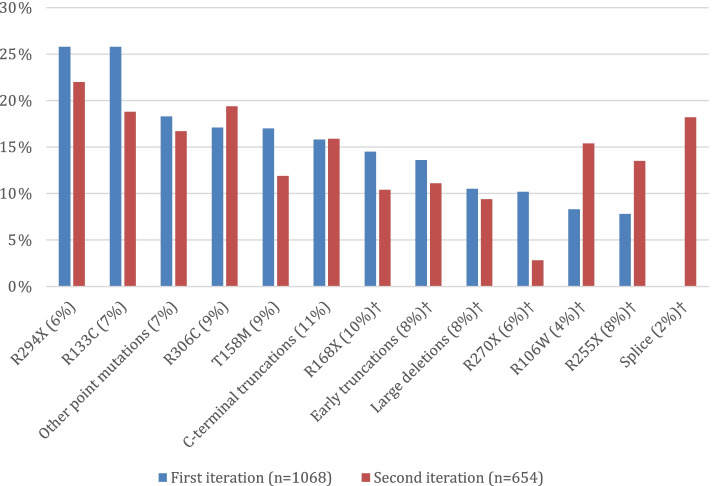
Frequency of anxiolytic use reported in medication logs by *MECP2* pathogenic variant. *MECP2* variant percent in parentheses indicates overall frequency of the variant in the entire study population. †Severe variant = R106W, R168X, R255X, R270X, early truncations, large deletions, and splice site. First iteration = subjects enrolled 2006–2015; second iteration = subjects enrolled 2015–2019

Review of medication logs found “anxiety” noted as an indication for a similar number of participants in both iterations of the RNHS: 15.0% of participants in the first and 14.5% in the second (*P* = 0.835). The most commonly used drugs were escitalopram, sertraline, fluoxetine, and buspirone. Anxiolytic treatment according to medication log entries did not vary by type of RTT diagnosis (typical or atypical) (*P* = 0.759). Participants on a medication for the indication of anxiety were older than their untreated counterparts (Table 3, Fig. 4). The proportion of participants on anxiolytic treatment was higher for participants over 18 years of age (21.3%) than for pediatric participants (12.9%; *P* < 0.001). Anxiolytic treatment was higher at younger ages in the first iteration than in the second iteration (Fig. 4). In the first iteration, anxiolytic use by medication log was also associated with lower clinical severity, but this was not replicated in the second iteration (Table 3). Similar to direct parental report, medication logs reported more anxiolytic use in participants with mild *MECP2* variants than severe variants in both first (20.1% vs. 10.9%, *P* = 0.001) and second (18.6% vs. 10.6%, *P* = 0.034; Fig. 5) iterations.

**Table 3:** Clinical features of participants based on treatment recorded in medication logs

Study	Feature	Any anxiolytic			SSRI		
		Yes	No	Significance	Yes	No	Significance
**First iteration (*n*= 1068)**	**Age (years)**	11.3 ± 9.7	8.7 ± 8.6	*P* < 0.000*	11.3 ± 9.8	8.76 ± 8.6	*P* < 0.000*
	**CSS total score**	19.9 ± 7.1	22.6 ± 7.8	*P* < 0.000*	18.9 ± 6.9	22.7 ± 7.8	*P* < 0.000*
	**MBA total score**	44.0 ± 14.1	47.4 ± 15.1	*P* = 0.006*	43.8 ± 14.0	47.4 ± 15.1	*P* = 0.006*
**Second iteration (*n*= 662)**	**Age (years)**	18.7 ± 10.6	14.6 ± 10.6	*P* < 0.000*	20.2 ± 10.6	14.5 ± 10.5	*P* < 0.000*
	**CSS total score**	24.0 ± 7.1	23.8 ± 8.5	*P* = 0.790	23.0 ± 7.2	23.9 ± 8.4	*P* = 0.324
	**MBA total score**	49.7 ±13.7	48.6 ± 13.8	*P* = 0.487	48.3 ± 10.7	48.8 ± 14.1	*P* = 0.710

Values are reported as mean ± standard deviation

*CSS* Clinical Severity Scale, *First iteration* Subjects enrolled 2006–2015, *MBA* Motor Behavioral Assessment, *Second iteration* Subjects enrolled 2015–2019

*P statistically significant

### Frequency of SSRI use by medication logs

Based on medication logs, SSRI use for any indication was reported for 13.1% of participants in the first iteration and 11.0% in the second iteration (*P* = 0.414). The indication for use of an SSRI in the second iteration was primarily anxiety (87%) and clinical findings were similar to the anxiolytic treatment group defined by medication logs. SSRI use was similar between participants with typical and atypical RTT (*P* = 0.383). SSRI use was associated with older age in both iterations of the RNHS, and with lower clinical severity in the first iteration (Table 3). SSRI use was more common in participants with mild than severe *MECP2* variants in the first (20.4% vs. 8.1%, *P* < 0.001) and second (15.9% vs. 8.0%, *P* = 0.018) iterations.

### Comparison of direct report and medication logs

Looking only at individuals in both studies (*n* = 352), the number of participants receiving anxiolytic treatment is similar whether the data were collected from medication logs in the first iteration (18.8%) or from direct report in the second iteration (18.5%; *P* < 0.000). In all participants in the second iteration (*n* = 662), anxiolytic treatment according to the medication log and to the direct report do not overlap perfectly (14.5% and 16.6%, respectively), but their relationship is significant (*P* < 0.001); this significance holds for participants either in both studies (*n* = 350, *P* < 0.001) or only in cohort 2 (*n* = 305, *P* < 0.001). The frequency of anxious/nervous behavior was directly related to anxiolytic treatment based on direct report and medication log report of anxiolytic or SSRI use (all *P* < 0.001).

## Discussion

Individuals with RTT display a wide variety of neurologic problems. Although the initial descriptions of the disorder highlighted autistic features, the current view is that other behavioral abnormalities are more significant and occur throughout the life of the individual. While several studies have reported on anxiety in RTT, these investigations have involved small numbers of participants and in general have not focused on this specific behavioral problem. The present study intended to provide the first large-scale examination of anxiety in RTT, utilizing the data collected by the multi-site RNHS. Because of the lack of records on psychiatric diagnoses in this study, we used a combination of parental report and medication logs from 1380 participants in two iterations of the RNHS. We confirmed the relatively high frequency of anxious or nervous behavior in RTT, which reached a level of clinical concern in at least 15% of participants as indicated by medication logs. We also identified frequency of anxiety-like behaviors, older age, and mild *MECP2* variants as predictors of or associated factors to anxiolytic and SSRI use in RTT. Although it was reported that pharmacological treatment achieved good control of anxiety in most participants, this behavioral problem represented a major concern for approximately 10% of caregivers.

Previous attempts to quantify anxiety-like behavior in RTT have relied on rating scales administered to parents/caregivers or clinicians. The Rett Syndrome Behaviour Questionnaire (RSBQ) is a parent/caregiver scale addressing problem behaviors in RTT, with a fear/anxiety subscale consisting of 4 items. Anderson et al. [1] reviewed the RSBQ scores of an Australian cohort of individuals with RTT (*n* = 137) and found that anxiety occurred “often” in 11% of participants and “occasionally” in 58%. This study similarly shows that a majority of participants’ parents consider them to be at least occasionally anxious or nervous, with a minority having more frequent anxiety-like behavior.

Taking into consideration the challenges in identifying and quantifying anxiety-like behaviors in RTT, we combined two sources of information to determine the frequency and severity of anxiety-like behaviors and their associated features in this population. Data from medication logs in the first iteration and direct reports in the second iteration were comparable, as was anxiolytic treatment according to medication logs between the first and second iterations. Data consistency was supported by comparing findings from participants in both studies with those only in cohort 2. As suggested by previous studies [1, 8, 19, 27], our analyses demonstrated a relatively high frequency of anxiety-like behaviors in RTT. The figure (~ 15%) is higher than that reported in the general pediatric population (~ 6.5% worldwide) [24]. However, compared to other neurodevelopmental disorders, anxiety in RTT is less common: ~ 80% in fragile X syndrome [10] and ~ 25% in autism spectrum disorder [26].

We evaluated severity of anxiety-like behaviors in RTT using a combination of measures, including parental selection of the most troublesome medical and behavioral issues affecting their child’s quality of life, behavioral problems requiring treatment, and the level of symptom control with treatment. Anxiety was one of the top quality of life concerns for a substantial proportion of participants (10%) and was the most common behavioral problem reported and treated, with reasonable control achieved in most cases (approximately two-thirds). To our knowledge, only one study has examined anxiety severity in RTT. A cohort of girls with RTT (*n* = 74) had moderate severity scores using the RSBQ and two other parent/caregiver scales that measure anxiety-like behavior in intellectual disability and autism spectrum disorder: the Anxiety, Depression, and Mood Scale (ADAMS) and the Aberrant Behavior Checklist–Community (ABC-C) [3]. This study also found that anxiety scores were inversely related to clinical severity and quality of life scores. In general terms, our study confirms the association between more severe anxiety and milder clinical severity. Both RNHS iterations demonstrated higher frequency and severity of anxiety-like behaviors in participants with milder *MECP2* pathogenic variants (R133C, R294X, R306C, and C-terminal truncations). However, the inverse relationship between anxiety scores and clinical severity was only present in the first iteration of the RNHS; it is unclear why this did not hold true in the second iteration.

In the RNHS, approximately 15% of participants used medications for anxiety. The present study was not designed to assess the efficacy or safety of these medications, but parent report indicates that most treated participants have good control. Associated factors to anxiolytic medication use in general, and SSRI use in particular, included increased frequency of anxiety-like behaviors, older age, and mild *MECP2* variants. Increased treatment in older individuals may indicate greater impact of anxiety on functioning at older ages, greater clinician comfort with prescribing these medications in an older population, or both. Interestingly, RTT diagnosis type (typical or atypical) was not related to frequency or severity of anxiety-like behaviors.

This study is based on a large cohort of 1380 females with RTT, with 352 participating in both iterations of the RNHS, studied over many years. Therefore, the results are likely to be generalizable to the broad RTT community. Males and individuals with RTT-like disorders were excluded from the analyses, so results presented here may not apply to these populations. In addition to children, we included information collected from adult participants in this study. However, older individuals in neurodevelopmental disorder research studies may be a non-representative group because of ascertainment bias (due to severity, caregiver motivation, or other factors). Clear limitations in this study include the lack of standardized questionnaires and objective measures of anxiety-like behaviors. In a phase 1 clinical trial of IGF-1 in RTT, improved right-sided alpha band asymmetry on electroencephalogram (EEG), a biomarker of anxiety and mood disorders, was mildly associated with lowered ADAMS scores in 5 out of 6 participants [16]. These data support the notion that anxiety-like phenomena are a component of RTT; however, adequate instruments for assessing anxiety in RTT are still lacking. The complex study design with a partially overlapping cohort added analytical challenges and limited the conclusions. Consequently, future anxiety research in RTT should focus on developing standardized clinical measures, such as questionnaires, and identifying biomarkers of relevance to this abnormal behavior. Follow-up studies should integrate clinical data, as the one analyzed here, with novel and already established biomarkers of anxiety in the general population (e.g., electrodermal activity, heart rate variability, cortisol, catecholamines). Complementing this, efficacy and safety of specific SSRIs and other anxiolytics should be carefully documented in the RTT population. Non-pharmacological treatments of anxiety should also be considered and evaluated in RTT. Ultimately, future studies should be focused on supporting improvements in the clinical care of anxiety in RTT, including the accuracy of diagnosis and effectiveness of treatment.

## Conclusions

This analysis demonstrates that most individuals with RTT display, at least occasionally, anxiety-like behavior and that anxiety is a significant parental concern. Older individuals and those with milder *MECP2* variants are more likely to be treated, and pharmacological treatment can improve symptoms. Better diagnosis and treatment of anxiety in RTT should be a goal of both future studies and clinical care.

## Data Availability

Data are housed at the NIH’s Rare Disease Clinical Research Network (RDCRN) Data Management and Coordinating Center, and are available through RDCRN’s data sharing policy. Data are also sent to the National Center for Biotechnology’s Database of Genotypes and Phenotypes annually.
